# DNA Barcoding Subtropical Aphids and Implications for Population Differentiation

**DOI:** 10.3390/insects11010011

**Published:** 2019-12-20

**Authors:** Qiang Li, Jun Deng, Cui Chen, Linda Zeng, Xiaolan Lin, Zhentao Cheng, Gexia Qiao, Xiaolei Huang

**Affiliations:** 1 State Key Laboratory of Ecological Pest Control for Fujian and Taiwan Crops, College of Plant Protection, Fujian Agriculture and Forestry University, Fuzhou 350002, China; 17750225044@163.com (Q.L.); tiancai1282008@126.com (J.D.); 17805963181@163.com (C.C.); lingdazeng@126.com (L.Z.); linxl@fafu.edu.cn (X.L.); zhentao.cheng0123@gmail.com (Z.C.); 2 Key Laboratory of Zoological Systematics and Evolution, Institute of Zoology, Chinese Academy of Sciences, Beijing 100101, China; qiaogx@ioz.ac.cn

**Keywords:** aphids, DNA barcoding, species identification, population divergence, phylogeny

## Abstract

DNA barcoding has proven its worth in species identification, discovering cryptic diversity, and inferring genetic divergence. However, reliable DNA barcode reference libraries that these applications depend on are not available for many taxonomic groups and geographical regions. Aphids are a group of plant sap sucking insects, including many notorious pests in agriculture and forestry. The aphid fauna of the subtropical region has been understudied. In this study, based on extensive sampling effort across main subtropical areas, we sequenced 1581 aphid specimens of 143 morphospecies, representing 75 genera, and 13 subfamilies, to build the first comprehensive DNA barcode library for subtropical aphids. We examined the utility of DNA barcodes in identifying aphid species and population differentiation and evaluated the ability of different species delimitation methods (automatic barcode gap discovery (ABGD), generalized mixed Yule-coalescent (GMYC), and Bayesian Poisson tree processes (bPTP)). We found that most aphid species demonstrated barcode gaps and that a threshold value of 2% genetic distance is suitable for distinguishing most species. Our results indicated that ten morphospecies may have species divergence related to factors such as host plant or geography. By using two pest species *Aphis spiraecola* and *A. gossypii* as examples, we also discussed the effect of the sampling scale of host plants on the results and reliability of DNA barcoding of phytophagous insects. This DNA barcode library will be valuable for future studies and applications.

## 1. Introduction

Since DNA barcoding was formally proposed at a large scale in 2003 [1], it has been widely used in different fields, for example species delimitation [2,3], cryptic diversity discovering [4,5], investigating host–parasitoid interactions [6,7], pest quarantine, and environmental monitoring [8,9,10]. A fragment of the mitochondrial cytochrome oxidase subunit I (COI) gene has been proven to be a standard DNA barcode region for animal species identification and detecting molecular operational taxonomic units (MOTUs) [11,12]. The usability of DNA barcoding has been reported in many groups, for instance Perciformes [13], invasive plants [3], Lepidoptera [2,4], Coleoptera [14,15], and Hymenoptera [16,17]. The power and accuracy of DNA barcoding, as well as unambiguous species identification may depend on sampling scales [18,19]. A previous study reported that the geographical scale of sampling has a critical impact on the performance and global application of DNA barcoding [20]. However, for many organisms including plant feeding insects, their species differentiation processes have been influenced not only by geography, but also by other factors (e.g., host plant for phytophagous insect). Theoretically, for a DNA barcoding project for phytophagous insects, the inclusion of samples from how many host–plant groups probably has an effect on the results and reliability of DNA barcoding analysis. Considering phytophagous insects occupy a large amount of species diversity on the Earth, investigation on this theoretical expectation about the sampling effect is necessary.

Aphids (Insecta, Hemiptera, Aphididae) are a group of small, plant sap sucking insects, with more than 5100 species all over the world [21]. This group includes many significant economic pests in agriculture and forestry, such as *Aphis gossypii* [22,23], *Myzus persicae* [24], *Schizaphis graminum* [25,26], and *Diuraphis noxia* [27,28], many of which have developed resistance to common insecticides [24,29]. In addition, aphids are notorious vectors of many plant viruses, such as soybean mosaic virus [30], cucumber mosaic virus [31], and pea mosaic virus [32], which can lead to tremendous damages and create huge economic losses. Based on our current knowledge, aphids can feed on more than 13,000 plant species, which belong to more than 300 plant families. Many aphids exhibit fidelity to their host plants, and host specialization has been proven to be critical for speciation and the evolution of species diversity [5,33]. Species identification of aphids is considered to be difficult due to morphological plasticity and sometimes a lack of obviously differentiated characteristics [34,35]. Traditional morphological identification requires high quality slides and specimens; however, a very small body size and complicated slide making procedure lead to a time consuming process of species identification, even for experts. Meanwhile, biotic factors such as life cycle [36], natural enemy [37], feeding site, and host plant [38,39], as well as abiotic factors [40] can also influence the morphological characteristics of aphids. A massive amount of intraspecific variation and phenotype similarity among different species could impede the identification of aphid species.

In recent years, the usability of DNA barcoding in aphid species identification has been tested in several studies [12,41,42,43,44,45]. In aphid species, researchers used DNA barcoding for exploring species diversity [45], identification of species with morphological ambiguity and detection of cryptic species [41,43,46,47,48], and even phylogenetic relationships [49,50]. Most of the DNA barcode sequences from previous studies or barcoding projects of aphids have been submitted to publicly available databases such as GenBank and the Barcode of Life Data Systems [51]. Previous studies of DNA barcoding of aphids were mainly confined to specific subfamilies or genera [46,50,52] or certain regions with a relatively small sampling [43,53]. However, considering there are more than 5100 known aphid species and many of them are widely distributed across the world [21], more DNA barcoding work of aphids is needed in order to build DNA barcode reference libraries, test the effectiveness of DNA barcoding for identifying aphid specimens in undersampled fauna (for example, subtropical region), as well as to reveal the cryptic diversity of aphid species.

As is well known, the total species diversity of many insect groups may decline from the tropics [54,55], such as in Lepidoptera [56]. Aphids are supposed to have originated in the temperate Northern Hemisphere [57,58], and their diversity in the tropics seems to be much lower than temperate zones [59], which displays a different pattern of latitudinal diversity gradient. Meanwhile, the subtropics located between the tropics and temperate zones have relatively higher environmental heterogeneity and species richness of plants, as well as insects [60,61]. Southern China has a typical subtropical climate and higher plant diversity, for which we think data and analyses of DNA barcode and aphid species diversity are important. The aphid fauna of this region has been understudied in history by comparison with temperate regions, leading to the lack of barcode data of some aphid groups mostly restricted to subtropical regions such as Hormaphidinae, Greenideinae, and Thelaxinae.

In the present study, based on extensive sampling across the main subtropical areas of southern China, a first DNA barcode reference library for subtropical aphids has been built. Based on this reference database, we also examined the utility of DNA barcoding in species identification, tested the accuracy of different species delimitation methods (automatic barcode gap discovery (ABGD), generalized mixed Yule-coalescent (GMYC), and Bayesian Poisson tree processes (bPTP)), and revealed several cases of the population differentiation of aphid pests.

## 2. Materials and Methods

### 2.1. Specimen Collection and Identification

In our study, aphid specimens were collected between 2015 and 2017 from ten provinces in southern China (Fujian, Yunnan, Zhejiang, Hainan, Guangxi, Guangdong, Shaanxi, Sichuan, Hunan, Jiangxi). Aphid individuals were preserved in 95% ethanol and stored at −20 °C for further molecular experiments. Detailed information of each specimen including collection locality, host plant, and GenBank accession numbers are listed in Table S1. Aphid samples were first identified morphologically based on reliable references and the taxonomic expertise of the authors. All voucher specimens were deposited in the Insect Systematics and Diversity Lab at Fujian Agriculture and Forestry University, Fuzhou, China.

### 2.2. DNA Extraction, Polymerase Chain Reaction, and Sequencing

The total genomic DNA was extracted from single aphid specimen using the DNeasy Blood &Tissue Kit (QIAGEN, Hilden, Germany). A nearly 700 base pair (bp) fragment of the cytochrome oxidase subunit I (COI) barcode region was amplified with the universal primers COI-LepF (5′-ATTCAACCAATCATAAAGATATTGG-3′) and COI-LepR (5′-TAAACTTCTGGATGTCCAAAAAATCA-3′) [12]. The 50 μL amplification cocktail contained 28.5 μL ddH2O, 8 μL dNTP, 5 μL 10× buffer, 2 μL of each primer (10 μM), 2.5 U of Taq DNA polymerase (5U/μL), and 4 μL of template DNA. The PCR conditions and processes were as follows: an initial denaturation step for 5 min at 95 °C, followed by 35 cycles consisting of 20 s at 94 °C, 30 s at 50 °C and 2 min at 72 °C, and final extension step for 7 min at 72 °C. The amplified PCR products were visualized in a 1% agarose gel and bidirectionally sequenced by Sangon Biotech (Shanghai, China).

### 2.3. Sequence Analysis

The BioEdit software [62] was used to revise any sequencing errors manually based on forward and reversed sequences. To confirm that the sequences were free of coamplification of nuclear mitochondrial DNA (Numt) or the mitochondrial orthologue in any endosymbionts, we also verified the sequences by checking whether they could be successfully translated into amino acid sequences without stop codons or frameshift mutations, as well as by running BLAST search. Subsequently, sequences were aligned by the MAFFT online version [63], then the output sequences were trimmed to the same length by using BioEdit. Finally, a matrix of sequences with a length of 560 bp was obtained for further molecular analyses. The haplotype sequences were extracted by DnaSP v5 [64]. For *Aphis spiraecola* and *A. gossypii*, two pest species with more specimens collected from various host plants, we conducted haplotype networks by using the NETWORK software [65] to further investigate their population differentiation patterns.

### 2.4. Genetic Distance and Phylogenetic Analysis

We calculated intraspecific and interspecific genetic distances using the Kimura 2 parameter (K2P) and prior intraspecific divergence (P) distance models [66] in MEGA 7.0 [67]. TaxonDNA [68] was used to construct the histogram to detect barcoding gap. MEGA 7.0 was utilized to construct the neighbor-joining (NJ) trees with the K2P model and 1000 bootstrap replicates. Considering all samples in our study belonged to Aphididae, three COI sequences of *Adelges cooleyi* from Adelgidae (Hemiptera) were used as outgroups (GenBank Accession Numbers KR045275, KR044627, KR044149). The layout of the NJ tree was edited by iTOL [69]. Furthermore, for *A. spiraecola* and *A. gossypii*, the genetic distances based on different host plant groups were also calculated to explore whether sampling effort related to host plant range had an effect on the reliability of DNA barcoding analysis.

### 2.5. Species Delimitation

In recent years, a number of methods based on molecular data have been proposed for delimiting species. In our analyses, three methods were used to assess species boundary and delimit possible species, including Automatic Barcode Gap Discovery (ABGD) [70], Generalized Mixed Yule-coalescent (GMYC) [71] and Bayesian Poisson Tree Processes (bPTP) [72]. ABGD, a clustering approach based on genetic distance for classifying different species, was performed on a web interface (http://wwwabi.snv.jussieu.fr/public/abgd/) with different models (JC69, K2P, prior intraspecific divergence (P) distance). The value of relative gap width (X) was set as 1.5, and prior intraspecific divergence (P) was set as 0.001–0.1. Combining the Yule model of species birth with the neutral coalescent model of intraspecific branching, the GMYC provides a robust method to define species boundaries [71]. An ultrametric tree was produced using the BEAST software [73] with the following settings: 200 million MCMC, log parameters every 20,000 generations, GTR + I + G model, strict clock, and coalescent: constant size. Then, the ultrametric tree was delimited with the SPLITS package (http://r-forge.rproject.org/projects/splits/) using the R program [74]. By assuming lower substitution numbers within a species than between species, the bPTP method calculates these two values in order to reach the aim of distinguishing species [75]. It uses a maximum likelihood (ML) phylogenetic tree instead of an ultrametric tree as input, which is simpler than GMYC. In our study, the model was selected based on the Bayesian information criterion (BIC) using jmodeltest [76]. The ML phylogenetic tree was constructed using raxmlGUI with the following settings: ML + rapid bootstrap, 1000 replicates, and the GTR + I + G model [77].

## 3. Results

### 3.1. Genetic Distances and Species Delimitation

A total of 1581 aphid specimens was successful sequenced in our study and identified as 143 morphological species, representing 13 subfamilies and 75 genera, collected from 244 host plant species in subtropical areas of southern China. These represented the first comprehensive COI DNA barcode reference library for subtropical aphids. The COI sequence data showed a strong bias toward A + T content (G + C = 23.3%, A + T = 76.7%). Haplotype analysis yielded 386 unique COI haplotypes in the whole dataset including outgroups (Table S1).

**Figure 1:**
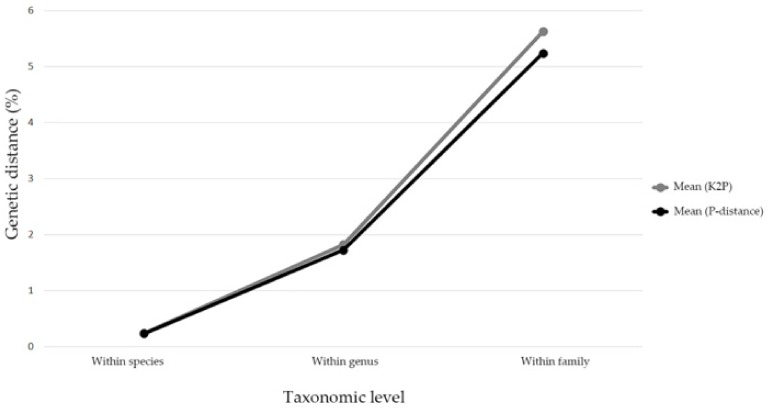
Line chart of mean genetic distances along with different taxonomic levels based on Kimura 2 parameter (K2P) and prior intraspecific divergence (P) distance models.

**Figure 2:**
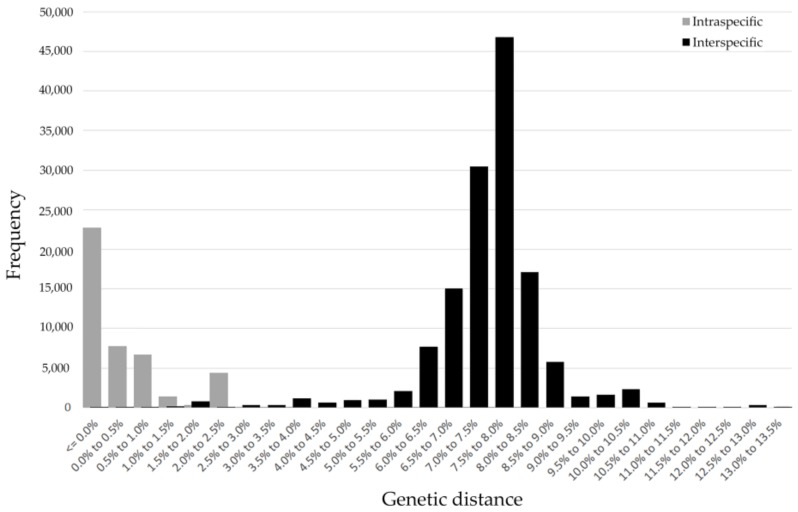
Frequency histogram of intra- and inter-specific (congeneric) genetic distances of subtropical aphids based on 1581 cytochrome oxidase subunit I (COI) sequences in our study.

The mean genetic distances were increased hierarchically alone with taxonomic levels based on K2P and P-distance models (intraspecific distance: 0.25% and 0.24%; congeneric distance: 1.83% and 1.73%; interspecific distance within families: 5.63% and 5.24%) (Figure 1). The genetic distances calculated by the K2P model were a little higher than the P-distances. Due to very similar results for the K2P and P-distance models, the K2P distances were used for further analyses. The intraspecific K2P distances ranged from 0 to 6.6%, and about 87.38% of the intraspecific distances were less than 2%, while 81.82% of the interspecific distances were more than 2%, which indicated an obvious barcoding gap in our data (Figure 2). Significantly high intraspecific divergences (2–6.6%) appeared within eight Aphidinae species (*Aphis aurantii*, *A. celastrii*, *A. gossypii*, *A. spiraecola*, *Hyalopterus pruni*, *Macrosiphoniella kuwayamai*, *Melanaphis sacchari*, *Sitobion avenae*), three species of Calaphidinae (*Shivaphis celti, Takecallis arundinariae*, *T. taiwana*), and two species separately from Chaitophorinae (*Periphyllus koelreuteriae*) and Neophyllaphidinae *(Neophyllaphis podocarpi*). The above species demonstrated clearly unique clades with two subclades in the NJ tree (Table S2, Figure S1). Meanwhile, low interspecific divergences (0–1.63%) were detected in *Melanaphis* (*Melanaphis sacchari* and *M. japonica*) and *Greenidea* (*Greenidea ficicola*, *G. psidii*, and *G. symplocosis*) (Table S2).

**Figure 3:**
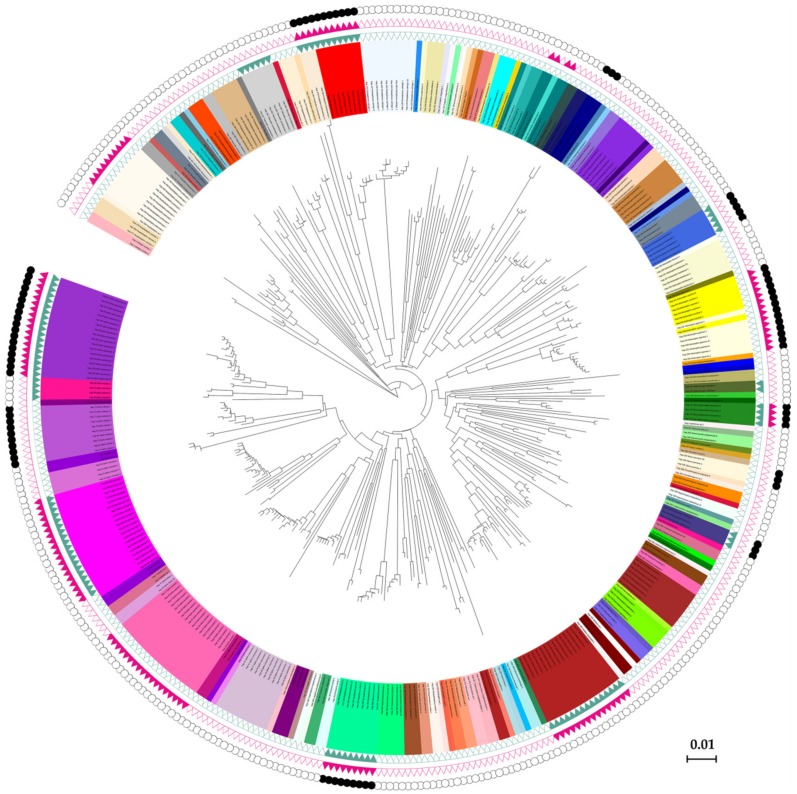
The species delimitation results in a circle plot style. The inner backbone tree shows the neighbor-joining tree based on COI haplotype sequences, and the inner circle with background colors shows different morphospecies. The three outside circles with green triangles, red triangles, and black dots represent the automatic barcode gap discovery (ABGD), generalized mixed Yule-coalescent (GMYC), and Bayesian Poisson tree processes (bPTP) results, respectively, on which the solid triangles or dots indicate the species having possible cryptic diversity.

Results based on ABGD, GMYC, and bPTP analyses were mostly concordant with the result of morphological identification (Figure 3). The analyses of ABGD with JC69, K2P, and P-distance were compared in our study. The number of groups based on the P-distance model ranged from 136 to 181, and initial partition engendered 154 groups (P = 0.0046–0.0129) (Table S3). Compared to the JC69 and K2P criteria, P-distance was close to the species number (143) of morphological identification and other species delimitation methods. The ABGD result showed that 13 morphological species had two different MOTUs (*Aphis spiraecola*, *A. celastrii*, *A. gossypii*, *Hyalopterus pruni*, *Macrosiphoniella kuwayamai*, *Matsumuraja rubifoliae*, *Brachycaudus helichrysi*, *Sitobion avenae*, *Shivaphis celti*, *Periphyllus koelreuteriae*, *Takecallis taiwana*, *T. arundinariae*, *Ceratovacuna keduensis*), while *Melanaphis japonica*-*M. sacchari* and *Greenidea ficicola*-*G. psidii*-*G. symplocosis* were obtained in a same MOTU, respectively (Figure 3).

The results of the GMYC analysis yielded 151 MOTUs, among which 127 were identical with the results of morphological delimitation. Two pairs of morphospecies (*Melanaphis japonica*-*M. sacchari* and *Greenidea ficicola*-*G. symplocosis*) were clustered in one clade, respectively. Ten species including *Aphis spiraecola*, *A. gossypii*, *A. aurantii*, *Hyalopterus pruni*, *Macrosiphoniella kuwayamai*, *Sitobion avenae*, *Shivaphis celti*, *Periphyllus koelreuteriae*, *Takecallis taiwana*, and *Neophyllaphis podocarpi* were observed in two independent MOTUs. For bPTP analysis, a total of 153 putative species was delimited. Those specimens from *Aphis spiraecola*, *A. odinae*, *Macrosiphoniella kuwayamai*, *Brachycaudus helichrysi*, *Myzus persicae*, *Shivaphis celti*, *Periphyllus koelreuteriae*, *Takecallis taiwana*, *Ceratoglyphina phragmitidisucta*, and *Ceratovacuna keduensis* formed two MOTUs, while *Hyalopterus pruni*, *Ceratoglyphina phragmitidisucta*, and *Ceratovacuna keduensis* showed three MOTUs. The two pairs of species (*Melanaphis japonica*-*M. sacchari* and *Greenidea ficicola*-*G. symplocosis*) showed an indistinct boundary in ABGD and GMYC, which was also obtained in bPTP analysis.

Considering that the result might be different from three species delimitation methods, a species with possible cryptic diversity was determined according to the following rule. If a morphospecies was shown with multiple putative species (molecular operational taxonomic units (MOTUs)) discovered by more than one method, then those species would be considered as having potential cryptic species. Our results indicated that there was a total of 10 morphospecies containing possible cryptic species (Table 1).

### 3.2. Species Divergence Related to Host Plant or Geography

**Figure 4:**
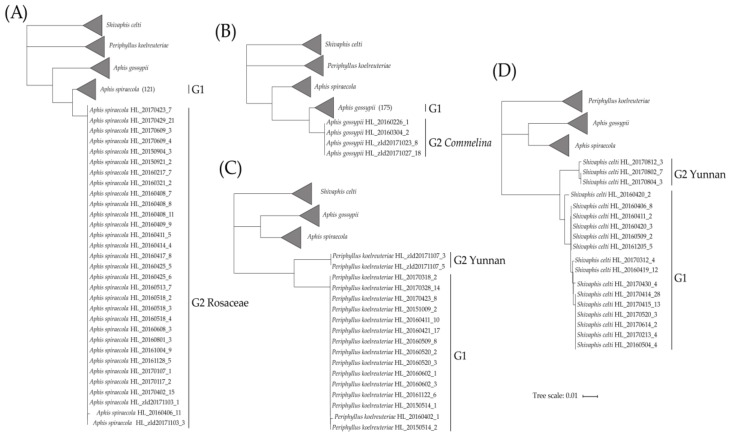
Simplified neighbor-joining cladograms of four species with obvious divergence, with each cladogram representing one species and the other three as outgroups. (**A**) *Aphis spiraecola*, (**B**) *A. gossypii*, (**C**) *Periphyllus koelreuteriae*, and (**D**) *Shivaphis celti*.

*Aphis* is the largest aphid genus with the greatest species diversity, which is widely distributed throughout the world [78,79]. A total of 16 morphospecies including 571 specimens of genus *Aphis* was analyzed in this study. One-hundred and fifty-two specimens of *A. spiraecola* were collected from 20 localities of the subtropical region and on 50 plant species of 23 plant families. NJ analysis revealed that there were two distinct clades (G1, G2) with a high bootstrap value; meanwhile, three delimitation methods showed the same results (Table 1, Figure 4A). The sequences of the *A. spiraecola* G1 clade were commonly from majority host plants and locations, while the sequences of the *A. spiraecola* G2 clade were almost restricted to Rosaceae plants. Significantly low intragenetic distances were observed within these two clades (*A. spiraecola* G1, 0–1.81%; *A. spiraecola* G2, 0–0.18%) compared to the relatively higher genetic distance between them (2.45%). As another example, *A. gossypii* with 179 specimens also showed similar results (Figure 4B). The genetic distances within the *A. gossypii* G1 and G2 clades ranged from 0 to 1.63% and 0 to 0.18%, respectively, while the genetic distance between the two clades was 2.93%. All the samples of *A. gossypii* G2 were collected from the host plants of *Commelina* (Commelinaceae). Deep intraspecific distance was also found between the two clades of *Periphyllus koelreuteriae* (n = 17; 6.6%; Figure 4C). The *P. koelreuteriae* G2 clade (0%) included two specimens from southwestern Yunnan, and fifteen specimens of the *P. koelreuteriae* G1 clade (0–0.2%) were collected from other southeastern areas of China. All specimens of them were from the plant genus *Koelreuteria* (Sapindaceae), which may indicate a role of geography in species divergence. Divergence was also discovered in *Shivaphis celti* (n = 18; 3.49%; Figure 4D), of which all specimens were collected on *Celtis* (Ulmaceae) from different geographical areas. However, three specimens from Yunnan formed the distinct *S. celti* G2 clade (0–0.4%), while the remaining specimens from southeastern areas formed another clade (0–1.3%).

### 3.3. Effect of Sampling Effort Related to Host Plant Coverage

**Figure 5:**
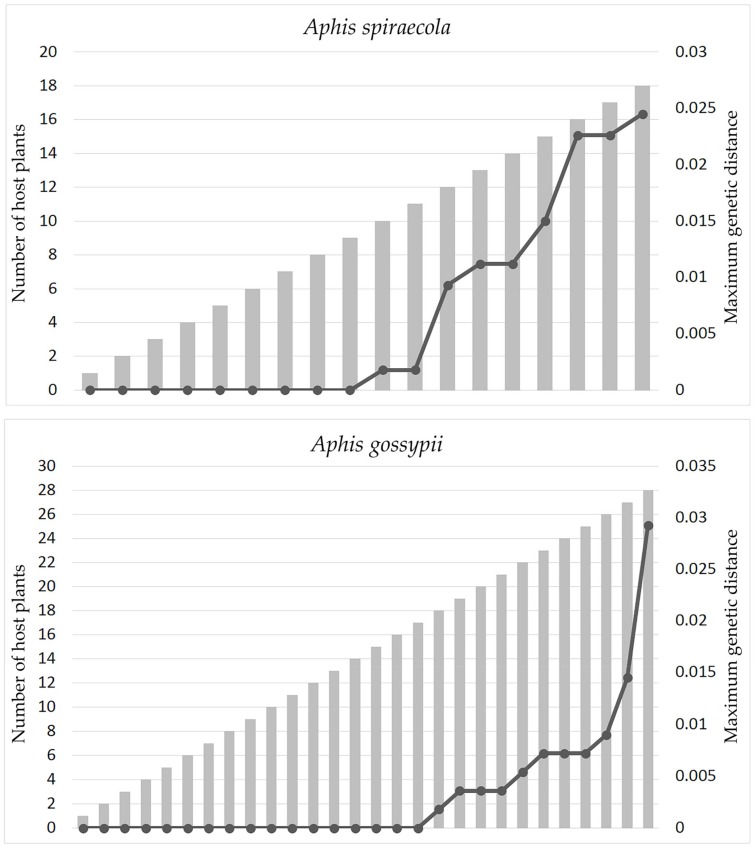
The distribution of intraspecific genetic distances of *Aphis spiraecola* and *A. gossypii* based on inclusion of samples from different host plant families. The gray bar indicates the number of host plant families, and the black line indicates the maximum intraspecific genetic distance.

**Figure 6:**
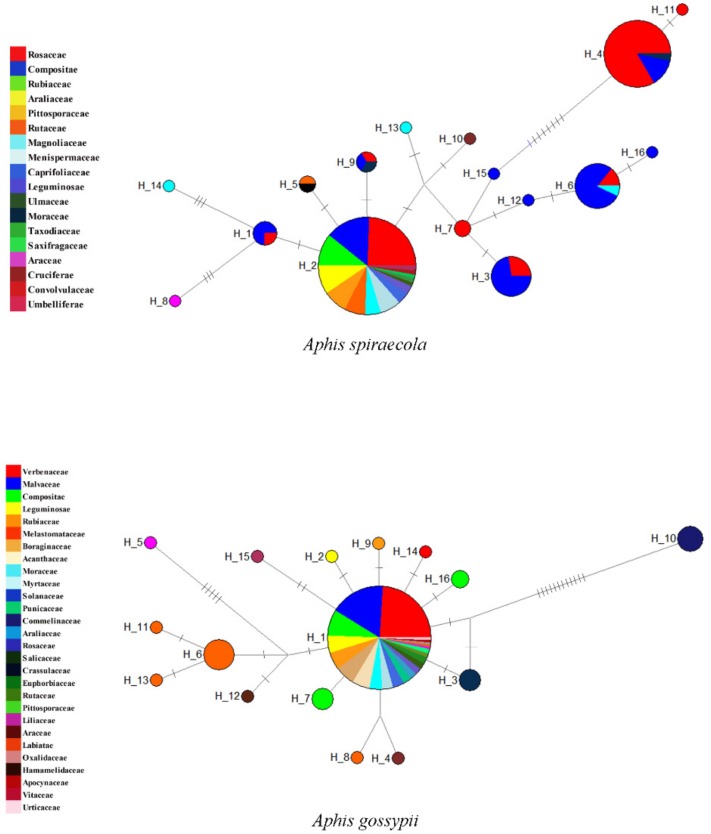
Haplotype networks of *Aphis spiraecola* and *A. gossypii* based on COI sequences grouped by host plant families. The circles represent different haplotypes, and the short line segments indicate mutated positions between haplotypes. Different colors and sizes of the circles represent host plant families and relative numbers of sequences (*A. spiraecola*: H_1: 4,H_2: 74, H_3: 11, H_4: 30, H_5: 2, H_6: 14, H_7: 2, H_8: 1, H_9: 3, H_10: 1, H_11: 1, H_12: 1, H_13: 1, H_14: 1, H_15: 1, H_16: 1; *A. gossypii*: Hap_1: 146, Hap_2: 1, Hap_3: 3, Hap_4: 1, Hap_5: 1, Hap_6: 6, Hap_7: 3, Hap_8: 1, Hap_9: 1, Hap_10: 4, Hap_11: 1, Hap_12: 1, Hap_13: 1, Hap_14: 1, Hap_15: 1, Hap_16: 2.

By using *Aphis spiraecola* and *A. gossypii* with sampling of a wide host plant coverage as examples, Figure 5 shows that the intraspecific genetic distances of the two species went up in proportion to the inclusion of more samples from different host plant groups. The haplotype network analyses of the two species based on samples grouped by host plant families (Figure 6) also indicated that haplotype divergences were usually related to host plant groups. Sometimes, the genetic divergence could be high, for example the *A. spiraecola* haplotypes from Rosaceae and Compositae and the *A. gossypii* haplotype from Commelinaceae. These results have important implications for DNA barcoding studies, which we discuss below.

## 4. Discussion

The molecular species boundary of many insect groups has been widely explored in the past. For examples, a 3.2% K2P genetic distance was set as a threshold value to distinguish different species in Carychiidae [80], while a 2% threshold value was used for delimitating intra- and inter-genetic variations in Lepidoptera [2], as recently reconfirmed by Zahiri et al. [81]. For different studies on the stink bugs in Hemiptera, their interspecific and intraspecific genetic distances also varied based on study groups and sampling [82]. In aphids, variable threshold values for species delimitation were used in different studies. A threshold value of 2% was used in two studies on Chaitophorinae [41] and Greenideinae [47], and a 2.5% threshold was used in a study on Calaphidinae [83]. For our current dataset, when the threshold value was set to 2%, the species partition based on genetic distance (133) was close to the species number (143) of morphological identification. In addition, most of the aphid species in our study demonstrated an obvious barcoding gap that the minimum interspecific distances were larger than the maximum intraspecific distances. Basically, DNA barcoding is a robust method to identify species rapidly and accurately [84,85] and discover possible cryptic species. Our research confirmed this in identifying aphid species from the subtropical region.

Several species delimitation methods based on DNA barcodes were proposed in recent years [70,71,72] and widely used in animal groups for recognizing species boundaries [41,43,86]. Several studies have shown that the MOUTs delimited by these methods sometimes were incongruent with each other [75,87]. Our results also showed slight difference among the MOUTs when ABGD, GMYC, and bPTP were used. ABGD showed 154 putative species with the highest MOUTs. Compared with ABGD, the advantage of GMYC and bPTP is that they include evolutionary information and rarely rely on the threshold of genetic distance [75]. However, some researchers suggested that GMYC usually overestimated species diversity [11,88]. In our study, GMYC analysis produced the most conservative result with 151 MOTUs and was more consistent with the number of morphospecies (143). The bPTP analysis is also a tree based method that imports a non-ultrametric phylogenetic tree [72]. Especially for some well defined species, it may be more accurate than GMYC [89,90]. The result of bPTP showed a medium number of MOTUs (153) in three methods. In general, the number of MOTUs based on three methods was very consistent in our study, indicating robust and credible species delimitation.

For phytophagous insects, the host plant usually plays an important role in their evolution and speciation [91,92]. With the development of the DNA barcoding technique [1], the discovering of cryptic species turned out to be easier than before [4,84]. Aphids feed and mate on their hosts, and the association with host plants can provide important information for species identification [46,47,50], and differentiation may occur by host shift and following specialization between intraspecific populations [5,93], even by feeding sites [94]. Some aphid species were described with overwhelming host specific lineages (host races) [5,93,95,96]. The selective pressure of host plants may play a significant role in aphid evolution and speciation [49]. *Aphis spiraecola* and *A. gossypii* are notorious pest species in agriculture and horticulture, with very wide host ranges [97,98], and therefore, divergences based on different host utilization may be more likely to occur within them. The differentiation of these two species has been less studied for subtropical populations. Our study found that both *A. spiraecola* samples associated with Rosaceae and *A. gossypii* samples associated with *Commelina* formed separate clades supported by all analyses. Furthermore, considering that there was not any evident divergence within these two species associated with geography, the population differentiation and possible cryptic diversity should be originated from host plant adaption. When looking at more species having genetic divergence and possible cryptic diversity indicated by our analyses, it seems that host plant related differentiation is common in aphids in the subtropical region, which may be related to the higher plant diversity in southern China.

The samples collected in southwestern Yunnan province of both *Shivaphis celti* and *Periphyllus koelreuteriae* formed separate clades (Figure 4C,D), and all three methods of species delimitation supported that *S. celti* and *P. koelreuteriae* may have cryptic species. These results indicate geography related differentiation in these two species. Yunnan is located in the mountains of southwest China and is a transitional region from the southern Himalayas to eastern Asia and from tropical Southeast Asia to subtropical southern China. The highly complex topography and broad altitude range spanning several thousand meters [99] lead to a unique vertical three-dimensional climate in this area, and shape one of the biodiversity hot spots in the world [100]. Cryptic species of other insects and plant groups were also reported in this area [101,102,103]. The geographical barriers in mountainous areas such as the southwestern mountains of China may obstruct genetic exchange with populations of other areas, resulting in genetic differentiation and possible speciation events [104,105]. As an example of geography related differentiation in hemipteran insects in another continent, high COI genetic distances (highest 4.7%) were observed between populations of stink bug *Chinavia hilaris* from the west coast and east coast of the United States and indicated that geographical isolation may promote cryptic diversity [82]. However, population differentiation of phytophagous insects may be driven simultaneously by both host plant and geography factors, as discussed in some previous cases [106,107]. Thus, more detailed studies on the differentiation of subtropical aphid populations at fine scales are needed in the future.

It has been reported that the geographical scale of sampling in DNA barcoding analysis has a critical impact on the performance and application of this technology [20]. We think that for phytophagous insects, especially those with a wide host plant range, the scale of sampling related to host plants should also have an effect on the results and reliability of DNA barcoding. Our study found that, within two aphid pests *Aphis spiraecola* and *A. gossypii*, the distribution of intraspecific genetic distance, as well as the haplotype divergence depend on the sampling scale of host plants (Figure 5 and Figure 6). There may be low genetic divergence among samples from several different host plant families, but distinct high divergence may exist between samples from certain host plants (e.g., *A. spiraecola* on Rosaceae and *A. gossypii* on Commelinaceae) and other plant families. This implies that, for phytophagous insects having a wide range of host plants, unbalanced and incomplete sampling may impede the performance of DNA barcoding analysis in species delimitation and uncovering population divergence. We suggest future DNA barcoding projects should take this sampling effect into consideration.

## 5. Conclusions

Based on extensive sampling across main subtropical areas of southern China, this study built a first comprehensive DNA barcode reference library for subtropical aphids, including 1581 COI sequences of 143 morphospecies representing 75 genera and 13 subfamilies. Three different species delimitation methods, the automatic barcode gap discovery (ABGD), generalized mixed Yule-coalescent (GMYC) and Bayesian Poisson tree processes (bPTP), yielded consistent results for our dataset, indicating robust and credible species delimitation. A threshold value of 2% genetic distance was suitable for distinguishing most species in this study. Ten morphospecies may have species divergence related to factors such as host plant or geography. By using *Aphis spiraecola* and *A. gossypii* as examples, we also revealed the effect of the sampling scale of host plants on the results and reliability of DNA barcoding of phytophagous insects. This study presents new information on aphid fauna of subtropical areas and the DNA barcode library will be valuable for future studies and applications.

## Figures and Tables

**Table 1:** The 10 morphospecies containing possible cryptic species indicated by more than one of the three delimitation methods (ABGD, GMYC, bPTP). The number of molecular operational taxonomic units (MOTUs), maximum intraspecific genetic distance, and collection information are provided.

Number of Cities	Number of Host Plant Species	Morphospecies	MOTUS			The Maximum Intraspecific Genetic Distance
			ABGD	GMYC	BPTP	
20	50	*Aphis spiraecola*	2	2	2	2.45
20	55	*Aphis gossypii*	2	2	1	2.93
6	8	*Hyalopterus pruni*	2	2	3	2.92
4	2	*Macrosiphoniella kuwayamai*	2	2	2	2.74
4	3	*Brachycaudus helichrysi*	2	1	2	1.81
5	7	*Sitobion avenae*	2	2	1	3.69
4	3	*Shivaphis celti*	2	2	2	3.49
10	5	*Takecallis taiwana*	2	2	2	3.69
3	2	*Ceratovacuna keduensis*	2	1	3	1.82
3	2	*Periphyllus koelreuteriae*	2	2	2	6.6

## Supplementary Materials

The following are available online at https://www.mdpi.com/2075-4450/11/1/11/s1: Figure S1: A neighbor-joining tree with bootstrap values based on all 1581 COI sequences of aphids from the subtropical region. The bootstrap values less than 75 are omitted. Table S1: Sample list and collection information of the 1581 specimens, including haplotype ID, specimen ID, host plant, and collection locality. KR045275, KR044627, and KR044149 are outgroups downloaded from GenBank. Table S2: The intraspecific and interspecific genetic distances of congeneric species of subtropical aphids. The genetic distances were calculated based on the K2P model. The intraspecific genetic distances were calculated when a species had at least two individuals. The interspecific genetic distances were calculated when a genus had at least two species. Table S3: Results of the automatic barcode gap discovery (ABGD) analyses.
